# Have one's view of the important overshadowed by the trivial: chronic progressive external ophthalmoplegia combined with unilateral facial nerve injury: a case report and literature review

**DOI:** 10.3389/fneur.2023.1268053

**Published:** 2024-01-05

**Authors:** Ziyang Feng, Rui Lai, Jia Wei, Xuan Liu, Xueqin Chen, Yangsicheng Liu, Wenxin Qin, Xiude Qin, Fanxin Kong

**Affiliations:** ^1^The Fourth Clinical Medical College, Guangzhou University of Chinese Medicine, Shenzhen, China; ^2^School of Acupuncture and Tuina, Chengdu University of Traditional Chinese Medicine, Chengdu, China; ^3^Department of Encephalopathy and Psychology, Shenzhen Traditional Chinese Medicine Hospital, Shenzhen, China

**Keywords:** chronic progressive external ophthalmoplegia, case report, misdiagnosis, muscle biopsy, genetic testing

## Abstract

Chronic progressive external ophthalmoplegia (CPEO) is a mitochondrial encephalomyopathy that is characterized by progressive ptosis and impaired ocular motility. Owing to its nonspecific clinical manifestations, CPEO is often misdiagnosed as other conditions. Herein, we present the case of a 34-year-old woman who primarily presented with incomplete left eyelid closure and limited bilateral eye movements. During the 6-year disease course, she was diagnosed with myasthenia gravis and cranial polyneuritis. Finally, skeletal muscle tissue biopsy confirmed the diagnosis. Biopsy revealed pathological changes in mitochondrial myopathy. Furthermore, mitochondrial gene testing of the skeletal muscle revealed a single chrmM:8469-13447 deletion. In addition, we summarized the findings of 26 patients with CPEO/Kearns–Sayre syndrome who were misdiagnosed with other diseases owing to ocular symptoms. In conclusion, we reported a rare clinical case and emphasized the symptomatic diversity of CPEO. Furthermore, we provided a brief review of the diagnosis and differential diagnosis of the disease.

## Introduction

In clinical settings, some atypical symptoms often misdirect doctors' attention during the diagnosis of some difficult diseases, thereby rubbing shoulders with the truth. For example, when chronic progressive external ophthalmoplegia (CPEO) is combined with other eye or facial symptoms, the diagnosis may be delayed for several years. CPEO is a subtype of mitochondrial encephalomyopathy that is characterized by a mutation in mitochondrial DNA (mtDNA) or nuclear DNA; this impairs adenosine triphosphate synthesis and subsequently leads to energy deficiency. Chronic progressive ptosis and impaired eye movement are the hallmark clinical symptoms of CPEO. In general, this disease occurs sporadically, with onset ranging from early childhood to ~50 years of age; nevertheless, it most often occurs before 30 years of age and affects both men and women at a similar ratio of ~1:1.8–2.5 (1, 2). Bilateral ptosis is often the initial symptom of CPEO; however, some patients may also experience diplopia and fatigue intolerance as initial symptoms. Progressive ptosis, ocular motility disorders, fatigue, and proximal limb weakness are the primary clinical manifestations of CPEO (3). However, the clinical presentation of CPEO overlaps with other conditions, including oculomotor myasthenia gravis, resulting in potential misdiagnosis. Herein, we present the case of a patient who was diagnosed after 6 years of seeking medical attention for her rare clinical presentation. Simultaneously, we briefly summarized the findings of previous patients with CPEO who were misdiagnosed with other diseases owing to their ocular symptoms.

## Case report

A 34-year-old woman presented with a history of incomplete left eyelid closure and limited eye mobility for 8 years; she had no relevant family history. In addition, she reported mild physical activity limitation; however, she did not promptly seek medical attention. Her parents are healthy non-blood relatives. In 2017, she visited an ophthalmology hospital for these symptoms; fundoscopy and optical coherence tomography revealed the absence of any anomalies. Subsequently, she was diagnosed with myasthenia gravis and prescribed oral pyridostigmine (60 mg tid) and prednisone (30 mg qd); however, her symptoms did not significantly improve. Prednisone dosage was gradually decreased to 15 mg QD. In 2018, she experienced fatigue and weakness 6 months after giving birth. In September 2019, she discontinued pyridostigmine and prednisone because she did not notice any substantial changes in her symptoms while on this medication. In late 2020, she developed left-sided facial hypoesthesia. In 2021, her limited eye movement worsened, with emotional stress during the 8th month of pregnancy, resulting in retesting and strabismus. She sought medical attention at another hospital, where she tested negative for anti-AchR, anti-MuSK, anti-Titin, and anti-VGCC antibodies. Routine biochemical tests and cerebrospinal fluid pressure were unremarkable. However, oligoclonal bands were observed in both blood and cerebrospinal fluid samples. Moreover, in both blood and cerebrospinal fluid samples, she tested negative for anti-AQP4, anti-MOG, anti-GFAP, and anti-MBP antibodies. Enhanced chest computed tomography (CT) did not reveal any thymoma. Furthermore, cranial magnetic resonance imaging showedrevealed no significant anomalies. Electromyography (EMG) results revealed the following: (1) mixed damage to the motor fibers of the temporal branch of the left facial nerve; (2) neurogenic injury of the left orbicularis oculi muscle; and (3) bilateral neurogenic injury of the quadriceps and tibialis anterior muscles. Finally, she was diagnosed with polyneuritis cranialis. Treatment with methylprednisolone pulse therapy and B vitamins was initiated; as a result, her strabismus and diplopia improved. She was discharged on oral prednisone (60 mg qd) and B vitamins; however, the symptoms of incomplete left eyelid closure and limited eye movement did not resolve.

When she was admitted to our department in September 2021, neurological examination revealed incomplete left eyelid closure and decreased sensation on the left side of her face. Furthermore, she exhibited limited eye movement in both eyes: there was significant upward, downward, outward, and inward movement in the left eye and significant upward, downward, and outward movement in the right eye. However, bilateral ptosis and nystagmus were not observed. The bilateral upper extremity muscle strength grade was 5, whereas the bilateral lower extremity muscle strength grade was 5-, with no muscle bundle tremor.

She gave birth to one son and one daughter. The eldest daughter seems to displaying signs of fatigue intolerance.

## Laboratory examination

At rest, the blood lactic acid levels were 1.8 mmol/L. After 15 min of exercise, lactic acid levels increased to 11.9 mmol/L; however, after 10 min of rest, they decreased to 8.1 mmol/L. The levels of serum lactate dehydrogenase (LDH), creatine kinase (CK), and creatine kinase isoenzyme (CK-MB) were in the normal range. Other blood routine and biochemical tests were unremarkable. Electrocardiography revealed mild changes in the T wave without any conduction block. EMG revealed the following: (1) electrophysiological signs of left facial nerve damage, characterized by lower wave amplitudes of action potentials in the orbicularis oculi, orbicularis oris, and nasal muscles compared with the contralateral side, with normal latency; and (2) negative electrophysiological findings in the repetitive electrical stimulation test. Brain magnetic resonance imaging revealed no anomalies. Finally, peripheral blood samples were collected for whole-exome and full-length mitochondrial gene sequencing. Genetic testing did not reveal any mutations in the relevant genes.

**Figure 1:**
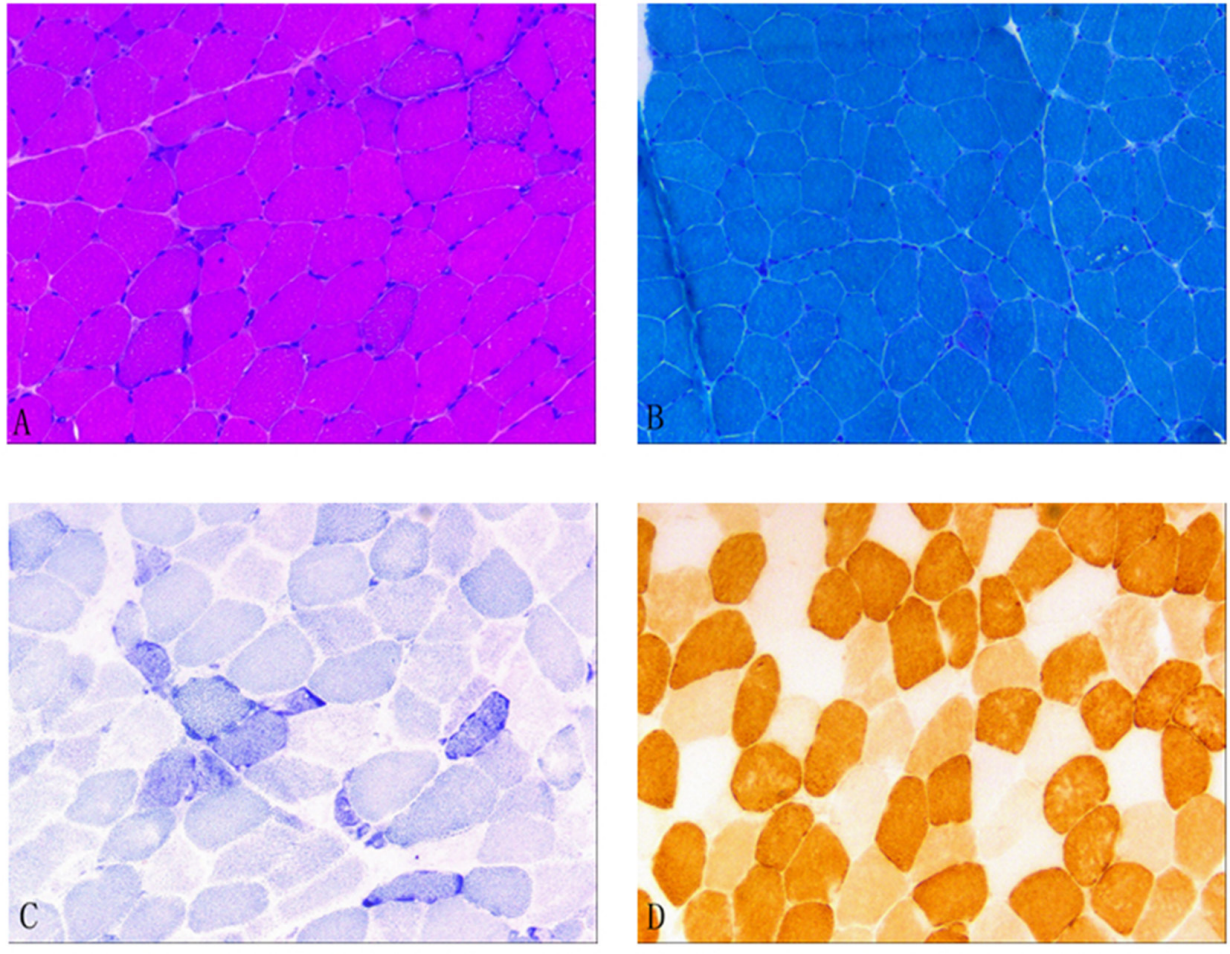
**(A)** Hematoxylin and eosin (HE) staining; **(B)** Modified Gömöri trichrome (MGT) staining; **(C)** Succinate dehydrogenase (SDH) staining; and **(D)** Cytochrome c oxidase (COX) staining. **(A)** HE staining revealed different muscle fiber sizes and scattered atrophic muscle fibers. **(B)** MGT staining revealed a few ragged-red fibers. **(C)** SDH staining revealed scattered ragged-blue fibers. **(D)** COX staining revealed several scattered negative muscle fibers.

**Figure 2:**
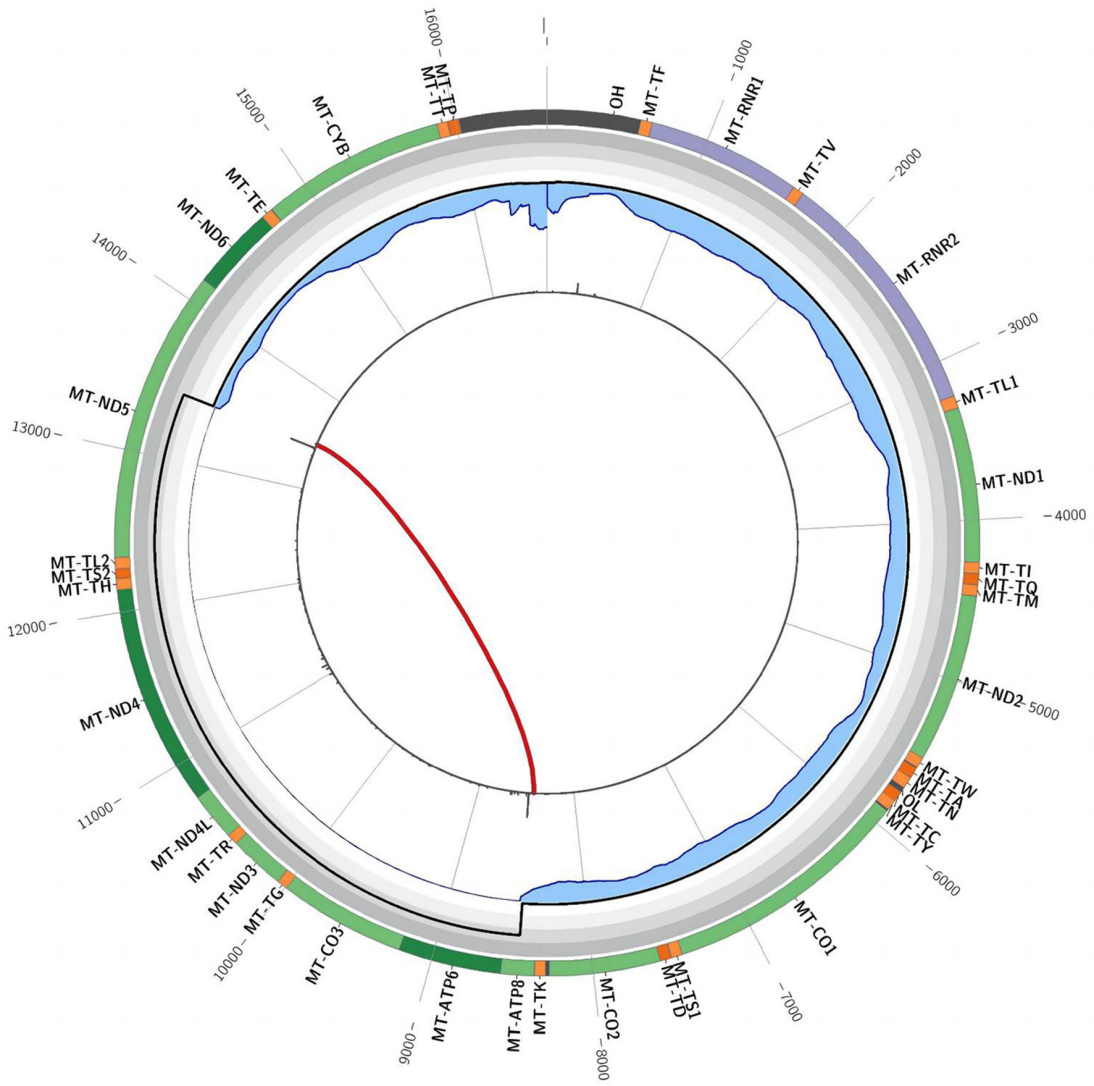
Triangular muscle mitochondrial gene testing. A single deletion (chrmM:8469-13447, arrow) was observed in the mitochondrial gene.

In December 2021, after obtaining consent from the patient, biopsy of the biceps muscle was performed for tissue analysis and genetic testing. Tissue biopsy revealed changes in mitochondrial myopathy. Furthermore, hematoxylin and eosin staining revealed muscle fibers of different sizes and scattered atrophic muscle fibers (Figure 1A). Modified Gömöri trichrome staining revealed a few ragged-red fibers (RRF) (Figure 1B). Moreover, succinate dehydrogenase (SDH) staining revealed scattered ragged-blue fibers (RBF) (Figure 1C). Cytochrome c oxidase (COX) staining revealed several scattered negative muscle fibers (Figure 1D). Although blood genetic testing did not reveal any relevant mutations, muscle genetic testing revealed the deletion of a meaningfully large fragment in the mitochondrial gene. Finally, mitochondrial gene analysis revealed the deletion of a large gene segment, with a single deletion region in chrmM:8469-13447. Based on these findings, the patient was finally diagnosed with CPEO (Figure 2).

## Discussion

CPEO is a rare disease that was first reported by Von Grafe in 1868. Studies have reported that the prevalence of CPEO differs in different populations. For example, a study published in *Neurology* in 2005 reported that the prevalence of large-fragment mtDNA deletions is 1.6/100,000 in adults in northern Finland (4). Another study reported that the minimum estimated prevalence of CPEO is 3.39/100,000 in the UK (5). CPEO can manifest at any age; however, it is often observed during childhood and adolescence, with a higher prevalence before 30 years of age. This condition can sporadically occur or run in families, indicating that it has both sporadic and familial forms. In general, CPEO initially presents with ptosis, with patients possibly experiencing diplopia and fatigue as their initial symptoms. Because no universally accepted diagnostic criteria for CPEO are available, a comprehensive approach involving clinical evaluation, laboratory tests, muscle tissue biopsies, and mitochondrial genetic testing is warranted to establish a diagnosis. Owing to symmetrical paralysis of the bilateral extraocular muscles and slow disease progression over months or years, compensatory mechanisms in the extraocular muscles frequently maintain a balanced ocular movement, resulting in infrequent complaints of diplopia or visits to the clinics for diplopia symptoms specifically (6, 7). In biochemical tests, the levels of CK, CK-MB, and LDH are frequently measured; these markers are generally normal or only mildly increased in patients with CPEO and serve as the initial screening indicators during diagnosis. Furthermore, blood lactic acid and pyruvate tests are vital for evaluating mitochondrial function. In some cases, blood lactic acid and pyruvate levels fail to return to normal within 10 min after exercise. However, in our case, only blood lactic acid was measured, without measuring pyruvate. Nevertheless, lactic acid test results aligned with the characteristic metabolic changes associated with CPEO.

Muscle tissue biopsy of the extremities is a valuable tool for diagnosing CPEO; it generally focuses on examining the proximal skeletal muscle. Necrotic and degenerated muscle fibers can be observed under a light microscope. Gömöri staining, which stains abnormal mitochondria, reveals the presence of RRF, which is a characteristic pathological feature of mitochondrial diseases. Furthermore, SDH staining, also called RBF staining, stains the muscle fibers blue; in general, it is a more sensitive method for detecting anomalies (8). COX staining can reveal COX(–) muscle fibers because respiratory chain enzymes are inhibited in mitochondrial diseases. In the present case, muscle tissue biopsy revealed the presence of RRF, RBF, and COX(–) muscle fibers; these findings were consistent with the characteristic histological changes associated with CPEO. Figure 1 provides a visual representation of the study findings.

In patients with CPEO, the primary mutation type is a single large-fragment deletion of mtDNA (9). A study conducted at Peking University First Hospital has revealed that the size of the deletion fragment of mtDNA is inversely correlated with the disease onset age, with larger deletions associated with earlier disease onset (10). Furthermore, point mutations in mtDNA or nuclear DNA can result in CPEO development (11, 12).

Mitochondrial genetic testing is the gold standard for CPEO diagnosis. Biological samples such as blood, urine, saliva, hair follicles, and muscle tissues can be used to perform genetic testing for mitochondrial encephalomyopathy. The first four samples can be collected non-invasively; however, muscle tissues achieve the highest positive rate, whereas blood samples achieve the lowest rate (13–16). Because mutant mtDNA is abundant in skeletal muscle tissues, they are generally preferred for testing purposes. A single major fragment deletion of mtDNA is commonly observed in patients with sporadic CPEO. Schon (17) identified a deletion fragment of 1.3 kb−7.6 kb, spanning from base 8483 to base 13459. In the present case, whole-exome sequencing of peripheral blood samples and full-length sequencing of mitochondrial genes only revealed mutations with no clinical significance. However, these findings did not align with muscle biopsy results; therefore, the patient underwent muscle tissue genetic testing. Fortunately, genetic testing confirmed the presence of CPEO (Figure 2).

Interestingly, in the present case, unilateral eyelid closure opacification and facial hypoesthesia were observed during the definitive diagnosis. EMG revealed facial nerve damage and neurogenic orbicularis oculi damage; therefore, the hospital diagnosed the patient with polyneuritis cranialis. This diagnosis was supported by improvements in the patient's symptoms after treatment with corticosteroids and B vitamins. Although CPEO is associated with peripheral neuropathy, it generally manifests as symmetric sensory and motor deficits in the limbs, rarely affecting the unilateral cranial nerves (2, 18, 19). Previous studies (20–22) have reported that the orbicularis oculi is involved in CPEO, resulting in incomplete closure of the eyelid. However, electromyography generally indicates myopathic weakness, with involvement of both sides. Furthermore, some studies (23, 24) have identified RRF in the muscle tissues of patients with CPEO and weakness of the orbicularis oculi via muscle biopsies, suggesting a myogenic lesion. Therefore, in the present case, the patient presented with a combination of unilateral facial nerve injury, which was supported by neurophysiological findings, and a possible trigeminal nerve injury, which remains unreported. Considering that incomplete eyelid closure did not worsen with the progression of oculomotor limitation in 2021, we suggest that the facial nerve injury in the present case was independent of CPEO. Subsequently, we believe that our patient experienced an independent left facial nerve injury during CPEO progression.

Owing to its rare clinical symptoms, CPEO can be easily overlooked or misdiagnosed. Therefore, differentiating it from other conditions that may present with similar extraocular muscle paralysis, including oculopharyngeal myasthenia gravis (OMG), oculopharyngeal muscular dystrophy (OPMD), and ocular pharyngeal distal myopathy (OPDM), is vital. Therefore, we reviewed the relevant literature on cases of CPEO/ Kearns–Sayre syndrome (KSS) that were previously misdiagnosed as other diseases owing to ocular symptoms (Table 1).

**Table 1:** Literature review of patients with CPEO/KSS who were misdiagnosed with other diseases owing to ocular symptoms.

**#**	**Sex**	**Onset age**	**Age at initial diagnosis**	**Age at final diagnosis**	**Clinicalfeatures**	**AchR**	**Serumlactate**	**LDH**	**CK**	**Muscle biopsy findings**	**Nucleotide changes**	**Previoustreatment**	**Previous diagnosis**	**Final diagnosis**	**References**
1	F	NM	35	56	Ophthalmoplegia and subjective muscle fatigue	Negative	NM	NM	NM	Possible mitocho ndrial cytopathy	NM	IVIG, AZA, pyridost igmine, and prednisone	MG	CPEO	(25)
2	M	7	8	15	Ptosis, exotropia, ophthalmoplegia, and weakness	Negative	NM	NM	NM	NM	m.6578-14460	Pyridost igmine	MG	KSS	(26)
3	M	57	58	61	Ptosis, diplopia, ophthalmoplegia, bradykinesia, and unsteady gait	Negative	NM	NM	NM	COX-negative fibers	POLG (c.2209G > A and c.3287G > A)	Pyridost igmine and levodopa	MG and possible progressive supranu clear palsy	CPEO	(27)
4	F	8	NM	10	Ptosis, weakness, decreased vision, and impaired hearing	Negative	NM	NM	NM	RRF	NM	NM	MG	KSS	(28)
5	M	30	NM	NM	Ophthalmoplegia, ptosis, weakness, dysarthria, and dysphagia	NM	NM	NM	Increase	NM	TWNK c.1361T > G	No treatment	OPMD	CPEO	(29)
6	F	adult	NM	NM	Ophthalmoplegia and ptosis	Negative	NM	NM	NM	RRF and COX-negative fibers	TWNK c.1070G > C	No treatment	MG	CPEO	
7	M	40	NM	NM	Ophthalmoplegia, ptosis, and weakness	Negative	Increase	NM	Normal	NM	TWNK c.1070G > C	Pyridost igmine	MG	CPEO	
8	F	60	NM	NM	Ophthalmoplegia, ptosis, weakness, and dysphagia	Negative	NM	NM	Normal	RBF and COX-negative fibers	TWNK c.1070G > C	Pyridost igmine and glucocor ticoids	MG	CPEO	
9	M	17	NM	NM	Ophthalmoplegia and ptosis	Negative	Normal	NM	Normal	COX-negative fibers	TWNK c.1121G > A	No treatment	MG	CPEO	
10	F	60	NM	NM	Ophthalmoplegia and ptosis	Negative	Normal	NM	Normal	RRF and COX-negative fibers	TWNK c.1361T > G	Pyridost igmine, acute episodes: IVIg, plasma exchange, and immunosu ppression	MG	CPEO	
11	M	25	NM	NM	Ophthalmoplegia and ptosis	Negative	Normal	NM	Increase	RBF and COX-negative fibers	TWNK c.1070G > C	No treatment	MG and OPMD	CPEO	
12	M	adult	NM	NM	Ophthalmoplegia and ptosis	NM	NM	NM	NM	RBF and COX-negative fibers	TWNK c.908G > A	No treatment	OPMD	CPEO	
13	F	40	NM	NM	Ptosis, weakness, and dysphagia	NM	NM	NM	NM	RRF and COX-negative fibers	TWNK c.1106C > T	No treatment	OPMD	CPEO	
14	M	40	NM	NM	Ptosis	Negative	NM	NM	Normal	RRF and COX-negative fibers	TWNK c.1070G > C	No treatment	MG	CPEO	
15	F	NM	NM	NM	Ophthalmoplegia	Negative	NM	NM	Normal	RRF and COX-negative fibers	TWNK c.1361T > G	No treatment	MG	CPEO	
16	F	adult	NM	NM	Ophthalmoplegia, ptosis, and weakness	NM	NM	NM	Normal	RRF	TWNK c.1361T > G	No treatment	OPMD	CPEO	
17	M	child- -hood	NM	NM	Ophthalmoplegia and ptosis	Negative	NM	NM	Increase	COX-negative fibers	TWNK c.1084G > C	No treatment	MG	CPEO	
18	F	29	34	34	Ophthalmoplegia and ptosis	Positive (ELISA) and negative (RIA)	NM	NM	NM	RRF	a single, large-fragment mitochondrial DNA deletion	Pyridost igmine	Possible ocular myasthenia	CPEO	(30)
19	F	40	41	43	Ophthalmoplegia, ptosis, arthralgias, proximal muscular weakness, and dysphonia	Negative	NM	Increase	Increase	RRF and COX-negative fibers	NM	Corticosteroids	Polymyositis	CPEO	(31)
20	M	40	64	64	Ophthalmoplegia, ptosis, neck weakness, quadriparesis, and dyspnea	Negative	Normal	NM	NM	RRF and COX-negative fibers	C10orf2 c.1433 G > T	Plasmapheresis, neostigmine, prednisone, and mechanical ventilation	Myasthenic crisis	CPEO	(32)
21	F	33	34	35	Ophthalmoplegia, ptosis, and weakness	NM	NM	Increase	Increase	RRF and COX-negative fibers	m.a deletion of 16.6 kilobases significant	Corticosteroid and cyclosporin A	Polymyositis	KSS	(33)
22	F	6	NM	25	Ophthalmoplegia, ptosis, weakness, and dysarthria	NM	Normal	Normal	Normal	Abnormal mitochondria	NM	NM	MG	CPEO	(34)
23	F	46	NM	52	Ophthalmoplegia, ptosis, and weakness	NM	NM	NM	NM	RRF and COX-negative fibers	NM	Anticholinesterases	MG	CPEO	(35)
24	M	14	16	29	Ophthalmoplegia, ptosis, diplopia, and weakness	NM	Increase	NM	Normal	RRF	NM	Anticholinesterases	Ocular myasthenia	CPEO	(36)
25	M	13	17	NM	Ophthalmoplegia and ptosis	NM	NM	NM	NM	NM	NM	NM	MG	CPEO	(37)
26	F	26	28	32	Ophthalmoplegia, incomplete left eyelid closure, exotropia, diplopia, and weakness	Negative	Increase	Normal	Normal	RRF, RBF, and COX-negative fibers	m.8469-13447	Pyridost igmine and glucocorticoids	MG and polyneuritis	CPEO	Our case

M, male; F, female; NM, not mentioned.

In total, 26 patients were misdiagnosed. Of these, 14 were females (53.8%). In 7 of 26 patients, the disease started when they were minors (≤ 18 years old); furthermore, 17 of 26 patients presented with symptoms in adulthood. For 2 patients, the age of disease onset was not mentioned.

The onset age was recorded in 20 patients, with a mean age of 31.55 ± 16.72 years. The age at first diagnosis was recorded in 10 patients, with a mean age of 33.5 ± 16.88 years. The age at final diagnosis was recorded in 12 patients, with a mean age of 38 ± 16.78 years. Considering the differences in the onset age between patients with KSS and CPEO, the mean onset age was 34.20 ± 15.47 years for 18 of 24 patients with CPEO, the mean age at first diagnosis was 36.33 ± 15.37 years for 9 of 24 patients, and the mean age at final diagnosis was 43.10 ± 13.42 years for 10 of 24 patients.

Oculomotor palsy was observed in 23 of 26 patients and ptosis was observed in 23 of 26 patients. Fatigue or malaise was observed in 15 of 26 patients. Strabismus was observed in 2 of 26 patients (case 2 and our patient). Diplopia was also observed in 2 of 26 patients (case 24 and our patient). Moreover, dysarthria or dysphagia was observed in 6 of 26 patients. Vision and hearing loss were observed in 1 of 26 patients, and arthralgia was also observed in 1 of 26 patients (case 19). Finally, 1 of 26 patients experienced unsteady gait and bradykinesia (case 3).

Of the 16 documented patients, 1 (case 18) tested positive for serum AchR antibodies via ELISA, resulting in the diagnosis of myasthenia gravis. However, clinical symptoms did not improve after 2 months of oral brompheniramine. After subjecting the initial serum samples to radioimmunoassay, the results were negative. This emphasizes the interference of test results in the presence of false positives in disease diagnosis.

Serum lactate was higher than normal in 4 of 8 patients. Furthermore, LDH was increased in 2 of 4 patients and CK was increased in 5 of 15 patients.

The muscle biopsy findings of 22 patients were reported, of which 20 exhibited RRF or/and COX(–) muscle fibers, whereas the remaining 2 patients had possible mitochondrial lesions (cases 1 and 22).

Furthermore, the genetic testing results of 19 patients were reported. Four patients had a single large-fragment deletion, whereas 15 patients had a point mutation.

Twenty patients were previously diagnosed with myasthenia gravis: 18 patients were diagnosed with ocular myasthenia and administered brompheniramine. On the other hand, five patients were previously diagnosed with OPMD, and two patients were previously diagnosed with myositis owing to increased LDH and CK levels (cases 19 and 21). Progressive supranuclear palsy owing to ataxia was suspected in one patient (case 3).

In general, OMG manifests as ptosis and diplopia, with milder symptoms in the morning, which worsen as the day progresses. OMG is commonly characterized by positive muscle fatigue and Tensilon test findings, the presence of serum anti-AChR antibodies, and positive repeated nerve stimulation test results. Furthermore, chest CT may confirm the presence of thymoma, and corticosteroid therapy is often effective. In our case, relevant antibodies and repeated nerve stimulation test results were negative, and chest CT revealed thymoma; therefore, OMG was excluded as a diagnosis.

OPMD frequently starts in middle age. Paralysis of the extraocular and pharyngeal muscles is an early clinical manifestation, with symptoms progressively exacerbating. Muscle pathology reveals rimmed vacuoles and fenestrated intranuclear inclusion bodies in the muscle fibers. Furthermore, genetic testing reveals mutations in *PABPN1*.

In general, the initial symptom of OPDM is droopy eyelids; however, it later progresses to dysphagia and limb weakness. Studies (38–41) have linked this condition to mutations in genes such as *LRP12, GIPC1, NOTCH2NLC*, and *RILPL1*. In the present case, we did not observe pharyngeal muscle weakness, and the genetic testing results were inconsistent with those of OPDM. Therefore, oculopharyngeal distal myopathy was excluded from the diagnosis.

KSS, a severe subtype of CPEO, is characterized by the risk of sudden death owing to heart block. It is defined using specific criteria (3, 42), including (1) onset before 20 years of age; (2) clinical features of CPEO accompanied by retinopathy pigmentosa; and (3) presence of one of the following: cardiac conduction anomalies, cerebrospinal fluid protein level > 1 g/L, or cerebellar dysfunction. In the present case, symptoms presented after 20 years of age, with no evidence of retinopathy pigmentosa. The protein levels in the cerebrospinal fluid were in the normal range, with no signs of ataxia or other types of cerebellar dysfunctions. Furthermore, electrocardiography did not reveal any conduction blocks. Therefore, our patient did not fulfill the diagnostic criteria for KSS at that time.

During the follow-up period until April 2023, incomplete left eyelid closure persisted, with limited movement of both eyes. Fortunately, there was no significant disease progression, and electrocardiography did not reveal any conduction block.

At present, no specific treatment modalities are available for CPEO, and medication primarily involves administering vitamins and coenzyme factors (43). Corticosteroids may help decrease lactic acid accumulation and provide therapeutic benefits for the disease. However, gene therapy holds promise as a potential treatment strategy for CPEO. Ongoing advances in gene therapy may lead to significant breakthroughs in the treatment of this disease in the future.

In the present study, we demonstrated the challenges faced by physicians in diagnosing CPEO, a rare presentation. The patient's clinical symptoms can mislead the physician's direction of diagnosis. Therefore, to diagnose such patients, physicians should master the diagnostic process as well as be familiar with the differential diagnosis of related diseases.

Our study has some limitations that should be acknowledged. Initially, the results of peripheral blood gene sequencing were not positive. We performed skeletal muscle biopsy only after a 3-month interval. Therefore, we suggest that patients with a clinical suspicion of mitochondrial encephalomyopathy, who can tolerate the pain associated with biopsy, should undergo skeletal muscle biopsy after directly consulting with them.

## Data availability statement

The original contributions presented in the study are included in the article/Supplementary material, further inquiries can be directed to the corresponding authors.

## Ethics statement

The studies involving humans were approved by the Shenzhen Traditional Chinese Medicine Hospital Ethics Committee. The studies were conducted in accordance with the local legislation and institutional requirements. The participants provided their written informed consent to participate in this study. Written informed consent was obtained from the individual(s) for the publication of any potentially identifiable images or data included in this article.

## Author contributions

ZF: Writing – original draft, Writing – review & editing, Supervision. RL: Writing – review & editing, Software. JW: Resources, Writing – review & editing, Investigation. XL: Resources, Writing – review & editing, Investigation. XC: Writing – review & editing, Investigation. YL: Writing – review & editing, Software. WQ: Investigation, Writing – review & editing. XQ: Writing – review & editing, Resources, Funding acquisition. FK: Writing – review & editing, Funding acquisition, Supervision, Writing – original draft.
