# Supplementary material for: Have one's view of the important overshadowed by the trivial: chronic progressive external ophthalmoplegia combined with unilateral facial nerve injury: a case report and literature review
Source: Front Neurol. 2024 Jan 5;14:1268053. doi: 10.3389/fneur.2023.1268053 (PMC10797034; doi:10.3389/fneur.2023.1268053)
Supplement: Supplementary file 1 [file Data_Sheet_1.PDF]

Nerve conduction velocity (NCV):

Nerve	Latency period (ms)	Amplitude (mV)	Segments	Distance (mm)	Speed (m/s)
**Facial.L**					
Orbicularis oculi	3. 8	2. 3			
Orbicularis oris	3. 4<br>U 4. 4	2. 1			
Nasalis	3. 9	1. 5			
**Facial.R**					
Orbicularis oculi	3. 2	2. 7			
Generic Muscle	3. 4<br>U 4. 4	3. 0			
Nasalis	3. 9	2. 2			
**Axillary.L**					
Supraclavicular foss	2. 4	7. 5	Deltoid-Supraclavicular fossa		
**Ulnar.L**					
Wrist	1. 9<br>U 4. 0	9. 3<br>L5. 00			
Belowel bow	4. 9	8. 6	Wrist - Below elbow	195	65<br>L47. 0
Above elbow	6. 0	8. 4	Below elbow- Above elbow	70	64<br>L47. 0

blink reflex (BR):

nerve	Stim side	Ipsi R1 - Lat ms	Ipsi R2 - Lat ms	Contra R2 – Lat ms	Diff R2 – Lat ms

Trigeminal.L

Left :	9.8	30.0	29.1	1.9
Right:	10.0	31.8	30.6	1.2
Difference:	0.2	1.8	1.5	

repetitive nerve stimulation (RNS):

Nerve **Amplitude** **Are**

Facial.R

10 stimuli at 3Hz

**times**	mV	decrement %	mVms	decrement %
1	0.81	0	3.30	0
2	0.81	0	3.32	-1
3	0.84	-4	3.30	0
4	0.89	-10	3.28	1
5	0.86	-6	3.30	0
6	0.87	-7	3.30	0
7	0.86	-6	3.34	-1
8	0.85	-5	3.31	0
9	0.86	-6	3.32	-1
10	0.88	-9	3.28	1

Facial.R

10 stimuli at 3Hz

1	2.11	0	1.81	0
2	2.13	-1	1.83	-1
3	2.15	-2	1.86	-3
4	2.15	-2	1.88	-4
5	2.14	-1	1.87	-3
6	2.15	-2	1.88	-4
7	2.15	-2	1.87	-3
8	2.17	-3	1.89	-4
9	2.15	-2	1.87	-3
10	2.15	-2	1.87	-3

10 stimuli at 3Hz

1	2.54	0	1.92	0
2	2.63	-4	1.91	1
3	2.68	-6	1.90	1
4	2.72	-7	1.89	2
5	2.71	-7	1.87	3
6	2.74	-8	1.88	2
7	2.74	-8	1.90	1
8	2.76	-9	1.90	1
9	2.75	-8	1.90	1
10	2.71	-7	1.87	3

Axillary.L

10 stimuli at 3Hz

1	6.35	0	36.20	0
2	5.62	11	31.40	13
3	5.82	8	32.20	11
4	5.40	15	29.10	20
5	5.70	10	31.10	14
6	5.93	7	32.50	10
7	5.85	8	32.40	10
8	5.80	9	32.50	10
9	5.95	6	32.80	9
10	5.29	17	28.90	20

Ulnar.L

10 stimuli at 3Hz

1	6.35	0	16.70	0
2	5.62	11	14.60	13
3	5.82	8	14.90	11
4	5.40	15	13.90	17
5	5.70	10	14.60	13
6	5.93	7	15.20	9
7	5.85	8	15.10	10
8	5.80	9	15.20	9
9	5.95	6	15.20	9
10	5.29	17	13.50	19

Ulnar.L

10 stimuli at 3Hz

1	8.50	0	17.60	0
2	8.47	0	17.70	-1
3	8.45	1	17.60	0
4	8.48	0	17.60	0
5	8.45	1	17.60	0
6	8.41	1	17.50	1
7	8.21	3	18.00	-2
8	7.92	7	18.50	-5
9	7.85	8	18.80	-7
10	7.79	8	18.90	-7
